# Development of a Web App to Enhance Physical Activity in People With Cystic Fibrosis: Co-Design and Acceptability Evaluation by Patients and Health Professionals

**DOI:** 10.2196/54322

**Published:** 2024-07-30

**Authors:** Raphaelle Ladune, Meggy Hayotte, Anne Vuillemin, Fabienne d'Arripe-Longueville

**Affiliations:** 1 Laboratoire Motricité Humaine Expertise Sport Santé Université Côte d'Azur Nice France

**Keywords:** cystic fibrosis, decisional balance, digital app, acceptability, physical activity, mobile phone

## Abstract

**Background:**

Cystic fibrosis (CF) is a genetic disease affecting the respiratory and digestive systems, with recent treatment advances improving life expectancy. However, many people with CF lack adequate physical activity (PA). PA can enhance lung function and quality of life, but barriers exist. The Cystic Fibrosis Decisional Balance of Physical Activity questionnaire assesses the decisional balance for PA in adults with CF, but it is not optimal for clinical use. A digital app might overcome this limitation by improving the efficiency of administration, interpretation of results, and communication between patients and health care professionals.

**Objective:**

This paper presents the development process and reports on the acceptability of a web app designed to measure and monitor the decisional balance for PA in people with CF.

**Methods:**

This study comprised two stages: (1) the co-design of a digital app and (2) the evaluation of its acceptability among health care professionals and people with CF. A participatory approach engaged stakeholders in the app’s creation. The app’s acceptability, based on factors outlined in the Unified Theory of Acceptance and Use of Technology 2, is vital for its successful adoption. Participants volunteered, gave informed consent, and were aged >18 years and fluent in French. Data collection was performed through qualitative interviews, video presentations, surveys, and individual semistructured interviews, followed by quantitative and qualitative data analyses.

**Results:**

In total, 11 health care professionals, 6 people with CF, and 5 researchers were involved in the co-design phase. Results of this phase led to the coconstruction of an app named MUCO_BALAD, designed for people with CF aged ≥18 years, health care professionals, and researchers to monitor the decisional balance for PA in people with CF. In the acceptability evaluation phase, the sample included 47 health care professionals, 44 people with CF, and 12 researchers. The analysis revealed that the acceptability measures were positive and that app acceptability did not differ according to user types. Semistructured interviews helped identify positive and negative perceptions of the app and the interface, as well as missing functionalities.

**Conclusions:**

This study assessed the acceptability of an app and demonstrated promising qualitative and quantitative results. The digital tool for measuring the decisional balance in PA for people with CF is encouraging for health care professionals, people with CF, and researchers, according to the valuable insights gained from this study.

## Introduction

### Background

Cystic fibrosis (CF) is a hereditary disease that mainly affects the respiratory tract and digestive system [1]. The disease is caused by a genetic mutation that affects the chloride transporter, a protein that regulates the water and electrolyte balance of cells. This mutation leads to the production of thick and sticky mucus that obstructs the airways, pancreatic ducts, and bile ducts [1].

Recent advances in CF management have drastically improved the life expectancy of people with CF [2,3]. Adult symptoms vary from cough and sputum to respiratory failure. Therapies aim to enhance patients’ quality of life: medications thin bronchial secretions, antibiotics treat infections, and enzymes aid digestion [4]. Innovative treatments such as gene therapy and cystic fibrosis transmembrane conductance regulator (CFTR) modulators show promise by correcting the malfunctioning protein produced by the CFTR gene [5]. Recent studies provided empirical evidence for improved survival rates, with an estimated median age of survival of approximately 50 years today [2]. However, the long-term management of adult patients with CF and the improvement in their quality of life are becoming increasingly important considerations.

### Physical Activity

Physical activity (PA) is crucial in the care of adults with CF [6]. PA includes sports, adapted PA, exercise, and recreational activities. It enhances lung function, fitness, and quality of life and lowers disease-related risks [7]. Importantly, PA is well tolerated, with no adverse effects in people with CF [8]. Recommendations for people with CF include aerobic exercise (eg, walking, jogging, cycling, and swimming) and strength training tailored to individual factors (eg, symptom variability, the initial level of physical fitness, preferences, and motivations) [9,10].

Many people with CF lack sufficient exercise, both in intensity and frequency [11]. Specific barriers to PA in adults with CF include physical (ie, fatigue and respiratory difficulties), psychological (ie, negative perceptions, a lack of motivation, and limited perceived ability), and environmental (ie, competing priorities, and a lack of opportunities and social support) barriers [12,13]. These barriers can be counterbalanced by facilitators of PA, including physical (ie, respiratory benefits, the improvement of overall health, and better preparation for transplantation), psychological (ie, positive perceptions of PA, improved self-esteem, and a sense of achievement), and environmental (ie, social support and the availability of sports facilities) facilitators [13].

The transtheoretical model of behavior change [14] outlines several stages: (1) precontemplation, (2) contemplation, (3) preparation, (4) action, and (5) maintenance. The decisional balance [15] reflects the balance between facilitators of and barriers to PA. This balance is important at each stage and may fluctuate due to external factors (eg, the availability of sports facilities and social support from peers) or internal factors (eg, personal motivation, energy level, and overall health status). In the specific context of CF, the Cystic Fibrosis Decisional Balance of Physical Activity (CF-DB-PA) questionnaire, described in Table 1, was developed and validated [16]. This 23-item tool indirectly measures the readiness of people with CF to change their PA level by measuring the individual’s facilitators of PA (ie, through 10 items divided into physical, psychological, and environmental factors) and barriers to PA (ie, through 13 items divided into physical, psychological, and environmental factors). A global score of decisional balance, as well as independent scores for each factor, can be calculated.

**Table 1:** Items of the Cystic Fibrosis Decisional Balance of Physical Activity (CF-DB-PA) questionnaire in French and English [16]^a^.

Number	Category	Items
1	F_PHY_^b^1	Cela développe mes muscles respiratoires et réduit mon essoufflement. (It develops my respiratory muscles and reduces my shortness of breath.)
2	F_PHY_2	Cela améliore mon endurance. (It improves my endurance.)
3	F_PHY_3	Cela améliore ma force et ma masse musculaire. (It improves my strength and muscle mass.)
4	F_PHY_4	Une bonne condition physique favorise la réussite de la greffe. (A good physical condition promotes transplant success.)
5	F_PSYCH_^c^1	C’est l’occasion de penser à autre chose. (This is an opportunity to think about something else.)
6	F_PSYCH_2	Cela me fait plaisir. (I am pleased to do it.)
7	F_PSYCH_3	Cela me permet de rencontrer d’autres personnes. (It allows me to meet other people.)
8	F_ENVI_^d^1	Je bénéficie d’un encadrement compétent pour ma pratique. (I benefit from competent supervision for my PA.)
9	F_ENVI_2	Je bénéficie de lieux adaptés à ma pratique. (I benefit from adapted places to do my PA.)
10	F_ENVI_3	J’ai une offre de pratique près de chez moi. (I have a PA offer in my immediate area.)
11	B_PHY_^e^1	Cela me fatigue trop. (It fatigues me too much.)
12	B_PHY_2	Je supporte mal l’effort physique. (I have trouble tolerating physical effort.)
13	B_PHY_3	Je m’essouffle très vite. (I get short of breath really fast.)
14	B_PHY_4	Je désature très vite. (I desaturate really fast.)
15	B_PSYCH_^f^1	J’ai peur d’être trop essoufflé.e. (I worry about getting short of breath.)
16	B_PSYCH_2	J’ai peur d’être contaminée par des germes dans les lieux de pratique sportive. (I am afraid of being contaminated by germs in places for PA.)
17	B_PSYCH_3	J’ai peur de tousser. (I am afraid of coughing.)
18	B_PSYCH_4	J’ai peur d’être mal vue si je tousse devant les autres. (I am afraid of being frowned upon if I cough in front of others.)
19	B_PSYCH_5	Je ne pense pas en être capable physiquement. (I don’t think I am physically able to do it.)
20	B_PSYCH_6	Je n’arrive pas à suivre le rythme. (I can’t follow the rhythm.)
21	B_ENVI_^g^1	Je n’ai pas le temps à cause de mes contraintes familiales. (I don’t have time because of my family obligations.)
22	B_ENVI_2	Je n’ai pas d’offre qui me convienne près de chez moi. (I don’t have a PA offer that works for me in my immediate area.)
23	B_ENVI_3	Je n’ai pas l’encadrement adapté à mes besoins. (I don’t have supervision that is adapted to my needs.)

^a^For each item, participants responded on a 6-point Likert scale ranging from 1 (“totally disagree”) to 6 (“totally agree”). The introduction sentence was “The factors that would encourage me to regularly practice a physical activity are...” (Les raisons qui m’inciteraient à pratiquer régulièrement une activité physique sont...) for the facilitators and “The factors that would hold me back from regularly practicing a physical activity are...” (Les raisons qui me freineraient à pratiquer régulièrement une activité physique sont...) for the barriers.

^b^F_PHY_: physical facilitator.

^c^F_PSYCH_: psychological facilitator.

^d^F_ENVI_: environmental facilitator.

^e^B_PHY_: physical barrier.

^f^B_PSYCH_: psychological barrier.

^g^B_ENVI_: environmental barrier.

This tool, developed in collaboration with CF centers, was initially well accepted by health care professionals and people with CF but has been poorly used since. The reasons given informally by the CF centers were mainly linked to the considerable amount of time needed to use it (ie, calculating each score is time consuming) and the limited perspectives for using it efficiently in clinical practice. Developing the CF-DB-PA questionnaire in digital form had the potential to meet these expectations. A previous review has already demonstrated the added value of digitalized questionnaires [17], which provide high levels of compliance, feasibility, and acceptability; improved data accuracy; and a reduction in data management and processing time. However, technical problems may occur, key factors of visual and ergonomic considerations must be taken into account, and the ease of using these questionnaires must be optimized [17]. It is, therefore, important to develop a digital format that is suitable for both health care professionals and people with CF, hence the interest in the co-design approach. Measuring the acceptability to the target population (ie, adults with CF and health care professionals) is usual in the process of technology development [18] and enables the user experience to be optimized. This paper presents the development process and reports on the acceptability of a web app designed to measure and monitor the decisional balance for PA in people with CF using the digital adaptation of the CF-DB-PA questionnaire.

## Methods

### Study Design

This study was conducted in two main stages: (1) the co-design of a digital app to define its content and (2) the evaluation of its acceptability among health care professionals and adult people with CF.

The development of the digital app was based on a participatory approach involving researchers and stakeholders (ie, people with CF and health care professionals) throughout the research process. This co-design methodology enables the production of useful and accurate knowledge that is consistent with the realities of key actors, culturally adapted to the target audiences, and logistically feasible to implement [19].

Acceptability is of paramount importance in the successful adoption and implementation of new technologies. This refers to the extent to which individuals perceive a specific innovation as useful, easy to use, and compatible with their values and needs. The Unified Theory of Acceptance and Use of Technology 2 (UTAUT2 [18]) explains and predicts individuals’ acceptability and use behaviors regarding new technologies. According to this model, acceptability is influenced by several key factors: performance expectancy, effort expectancy, social influence, facilitating conditions, habits, price value, and hedonic motivation.

Numerous studies have applied and validated the UTAUT2 model in various contexts, including health care, education, and organizations [18,20]. Understanding and considering the factors that influence acceptability can assist designers, decision makers, and practitioners in enhancing the adoption and use of innovations, thereby promoting more successful and sustainable changes.

### Participants

Participants in the co-design phase included health care professionals specialized in CF, people with CF, and researchers. Objectives of this co-design phase (ie, to build a prototype of the app to adapt the CF-DB-PA questionnaire in digital form) were presented to participants. Health care professionals and people with CF were recruited from 2 specialized centers in France selected for their advanced PA promotion practices. Researchers were specialized in sports sciences, PA promotion to people with CF, or patients with other conditions. They were selected from 2 French universities. Participants were approached via email and phone calls.

Participants in the acceptability evaluation phase included people with CF, health care professionals specialized in CF, and researchers specialized in sports sciences. Objectives of this acceptability phase (ie, to understand perceptions of the first version of the app) were presented to participants. Participants were approached face to face or via email or phone call. No relationship was established before the start of the study. After survey completion and when participants were available for 30 more minutes, a semistructured interview was proposed.

Participants in the co-design phase were allowed to participate in the acceptability phase. All were aged >18 years old and fluent in French.

### Data Collection

#### Co-Design of the Digital App

The co-design of the digital app was divided into several steps. First, qualitative individual interviews were conducted with people with CF and health professionals. These interviews explored the perceptions of the CF-DB-PA questionnaire to promote PA among people with CF. The interview guide was composed of four parts: (1) current professional practices related to PA, including initial patient encounters, the assessment of the patient’s PA, counseling, and orientation toward PA; (2) perceptions of the CF-DB-PA questionnaire, including perceived benefits, identified limitations, and potential uses; (3) the functionalities that could be added to the digital version of the questionnaire; and (4) the prospects for using the digital app. Interviews were conducted by the first author (a female PhD student in human movement sciences), who was trained to conduct semidirective and focus group interviews. The interviews were audio recorded, and notes were taken.

On the basis of the results, the authors brainstormed a first preprototype of a digital app designed to measure and monitor the decisional balance for PA in people with CF. Then, 3 consecutive meetings were organized by the first author alone with all the health care professionals, people with CF, and researchers involved in the co-design phase to achieve a prototype. During the first meeting, the operational aspects of the digital form of the CF-DP-PA questionnaire were identified. The second meeting focused on listing the functions to complement the CF-DB-PA questionnaire administration. During the third meeting, the specifications were classified according to their importance into 3 levels: priority, secondary, or tertiary. The second and third meetings were introduced with a summary of the previous meeting. An overall written summary was sent to the participants after the last meeting, listing the elements that needed to be included in the digital version of the questionnaire.

#### Acceptability of the Digital App

##### Overview

The acceptability of the first version of the app was assessed individually by a wider group of the 3 types of participants. Participants were presented with a narrated video showing and explaining the different functionalities of the app, including (1) an introduction to the definition of decisional balance and the barriers to and facilitators of PA among people with CF and (2) the functionalities of the app from the perspectives of people with CF, health care professionals, and researchers. After watching the video, participants were invited to complete a survey administered by LimeSurvey (Lime Survey GmbH, Version 3.17.3+190429) in the CF centers or through videoconferencing; the survey comprised several questionnaires.

##### Acceptability of the App

The French eHealth scale based on the UTAUT2 [21] was specifically adapted to the presented digital app (ie, “CT for Health” was replaced by “this app”) and used. This scale is composed of 25 items divided into eight factors: (1) performance expectancy, (2) effort expectancy, (3) social influence, (4) facilitating conditions, (5) hedonic motivation, (6) price value, (7) habit, and (8) behavioral intention. Items were evaluated on a 7-point Likert scale ranging from 1 (strongly disagree) to 7 (strongly agree). The Cronbach α coefficients were high [22], as they ranged from 0.74 to 0.94.

##### Use Frequency of Technology-Based PA Interventions

On the basis of the original UTAUT2 questionnaire [18], questions on the actual use of technology-based PA interventions (ie, mobile apps, sensors, social media, videos, and active video games) were asked. The use frequency of each technology was evaluated on a 7-point Likert scale as follows: (1) never, (2) less than once a year, (3) once a year, (4) once a month, (5) once a week, (6) once a day, and (7) several times a day. The Cronbach α coefficient was satisfactory [22], with a value of 0.70.

##### Computer Anxiety

Four items of the computer anxiety trait subscale [23] were used. These items were selected based on their relevance to the study context. Computer anxiety was measured on a 5-point scale ranging from 1 (not at all corresponding) to 5 (completely corresponding). The items presented high internal consistency (ie, Cronbach α coefficient=0.93) [22].

##### Sociodemographic Information

Questions concerning age, sex, socioprofessional category, and CF mutation type were asked.

In the second step, participants were asked whether they would agree to participate in complementary, individual, and semistructured interviews. The interview guide comprised four parts: (1) perceptions of the app, (2) perceptions of the interface, (3) missing functionalities, and (4) overall feedback and app use intentions. Interviews were conducted face to face by the first author at specialized CF centers after the completion of the survey or remotely through videoconferencing. The interviews were audio recorded, and notes were taken. A summary was produced at the end of the interview to validate the participant’s statements.

### Data Analysis

#### Co-Design of the Digital App

The interviews were analyzed based on the core principles of thematic analysis [24-26]. First, the audio recordings of the interviews were transcribed verbatim. Second, the transcripts were read multiple times by the first author and a second person (mentioned in the acknowledgments) to gain a thorough understanding of the data. Deductive coding in the previously defined main categories was initially performed by them, followed by inductive coding in an Excel spreadsheet (Microsoft Corp). The last 2 authors checked each verbatim coding and its classification. They had access to the original transcripts to contextualize the review of the coding. The first author and the last 2 authors discussed the codes and classification until consensus was reached. The second author acted as a disinterested peer [27] without access to the original transcripts. All researchers then discussed the coding until consensus was reached. To ensure methodological rigor, criteria were reported according to the COREQ (Consolidated Criteria for Reporting Qualitative Research) guidelines [28].

#### Acceptability of the Digital App

All analyses were performed using SPSS (version 23; IBM Corp). The sample had no missing data (web completion of the survey). The data were first analyzed through descriptive methods (means and SDs). Multiple regression analyses were then used to examine the explained variance and the main contributors to the behavioral intention to use the app prototype. Differences between the dependent variables (ie, UTAUT2 dimensions, frequency of use of technology-based PA interventions, and computer anxiety) according to participant type (ie, health care professionals, patients, and researchers) were examined using multivariate and univariate analyses of variance tests.

Qualitative interviews were analyzed using the same methodology as the one previously described for the digital app co-design phase. To ensure methodological rigor, criteria were reported according to the COREQ guidelines [28].

### Ethical Considerations

The Research Ethics Committee of the University of Côte d’Azur approved this observational study (CER 2022-049). All participants were volunteers and gave informed consent. The anonymity and confidentiality of participants and data were guaranteed in accordance with the General Data Protection Regulation. We ensured that no identification of individual participants was possible in the reported data and quotes. The interviews were transcribed without personal information and were deleted after transcription. No compensation was paid to participants.

## Results

### Co-Design Development of the App’s Content

#### Characteristics of the Sample

In total, 11 health care professionals specialized in CF (physicians: n=2, 18%; coordinating nurses: n=2, 18%; physical therapists: n=2, 18%, adapted PA professionals: n=3, 27%; psychologist: n=1, 9%; and dietitian: n=1, 9%), 6 people with CF, and 5 researchers (specialized in health psychology and PA promotion) participated in the co-design phase. The health care professionals (male: n=3, 27%; female: n=8, 73%) had a mean age of 39.91 (SD 11.60) years and took part in the individual interviews and the 3 co-design meetings. The 6 people with CF (male: n=2, 33%; female: n=4, 67%) had a mean age of 28.17 (SD 6.94) years. They carried the most common genetic mutation, which gave them access to CFTR modulator treatment. Their degrees of disease severity were classified as “average.” They took part in the individual interviews and the 3 co-design meetings. No participant dropped out of the study or refused to take part.

The interviews were conducted from January 2022 to May 2022, totaling 691 minutes (individual interviews: 560 min, mean 32.94, SD 4.46 min; focus group: 131 min, mean 43.67, SD 2.52 min). The interviews were ended when no new data were provided by the participants and a consensus was reached about emerging themes.

#### Outputs From Co-Design Development Interviews

##### Overview

On the basis of the interview guide, the following four categories were studied: (1) current professional practices related to PA, (2) perceptions of the CF-DB-PA questionnaire, (3) the functionalities that could be added to the digital version of the questionnaire, and (4) the prospects for using the digital app. Examples of quotes representing these categories are provided in Table 2.

**Table 2:** Representative quotes from the co-design development of the app’s content with health care professionals (n=11) and people with CF^a^ (n=6).

Themes and categories				Participants, n (%)		Quotes
**Professional practices in PA^b^ management (n=11, 65%)^c^**						
	First encounter with people with CF		11 (100)		Quote 1: “At every visit, we have at least one question about what we’re doing [in PA], how it’s going, and so on, we always have something to say about PA practice” (person with CF #3).	
	Assessment of the PA of people with CF and referrals		11 (100)		Quote 2: “Yeah, we’re encouraged to do a sport, but we can choose, and the good thing is that here [in the specialist center] they show us several, so at least we can try and see what we like and don’t like” (person with CF #1).	
**Perceptions of the CF-DB-PA^d^ questionnaire (n=17)**						
	**Benefits of the CF-DB-PA** **(n=17, 100%)**					
		Individualized results	9 (53)^c^		Quote 3: “It lets us get to know our patients better, to see things that come to light that we wouldn’t necessarily have identified, such as their motivations.” (adapted PA professional #3).	
		Evaluation of motivation levels	7 (41)		Quote 4: “It might also be good to see who’s sports-oriented and likes it, and who’s not so keen on it” (psychologist #1).	
		Understanding the knowledge of people with CF	7 (41)		Quote 5: “This will challenge us doctors, our misconceptions, what we think is obvious or taken for granted [by our patients] when it isn’t” (physician #1).	
		Encourages PA practice	7 (41)		Quote 6: “Just the fact that the questionnaire is on display is a plus, it makes you question yourself, and why wouldn’t it encourage you to start exercising again?” (nurse #2)	
	**Limitations of the CF-DB-PA** **(n=15, 88%)**					
		Time consuming	13 (76)		Quote 7: “Consultation time is already sometimes not enough, so taking the time to carry out the analyses correctly seems very complicated to me” (adapted PA professional #1).	
		Paper-based format	9 (53)		Quote 8: “In the department, we try to reduce the use of paper as much as possible, and now it would be even harder to scan it and include it in the patient’s file. It would take even more time, and I don’t think anyone would want to take that on” (physical therapist #1).	
**The app’s contents (n=17, 100%)**						
		User-friendly interface	15 (88)		Quote 9: “It [the app] really needs to be simple so that people understand straight away and don’t waste time looking for where to go” (person with CF #4).	
		Easy data extraction	11 (65)		Quote 10: “We need something that we can retrieve and put in the patient’s individual file, without having to print and scan it” (dietitian #1).	
		Secure patient data handling	8 (47)		Quote 11: “It still has to be something serious, so that not just anyone can log on and see our answers and even our personal information” (Person with CF #2).	
**Prospects for using the digital application (n=1, 6%)**						
	Willingness to adopt the digital format		1 (6)		Quote 12: “It’s true that I’m not necessarily a big fan of anything connected. So personally I don’t think I’ll be using it” (person with CF #5).	
	**Reasons to adopt the digital format** **(n=16, 94%)**					
		Limit the use of the paper format	14 (82)		Quote 13: “Completely, because here we really have the objective, I think like everywhere else, of doing more and more without paper, so yes, if there was an application, we would use it all the more” (adapted PA professional #2).	
		Increase in the use of smartphones	10 (59)		Quote 14: “We have quite a few patients from a generation that is fairly positive about apps and things like that, so I think it could go down very well” (physician #2).	

^a^CF: cystic fibrosis.

^b^PA: physical activity.

^c^Health care professionals only.

^d^CF-DB-PA: Cystic Fibrosis Decisional Balance of Physical Activity.

##### Professional Practices in PA Management

This category was divided into two subcategories: (1) first encounter with people with CF and (2) assessment of the PA of people with CF and referrals. During the initial consultations, health care professionals systematically promote PA to people with CF, starting from early stages of care. They conduct follow-ups during quarterly visits, emphasizing the benefits of PA and the various activities available at specialized centers. Advice focuses on safety, hygiene, nutrition, hydration, and functional capacity rather than performance. Support extends beyond hospitals, with respiratory physiotherapy as a common thread. A few tools, including pedometers and existing therapeutic education tools, are used to promote PA. Patients emphasized the importance of PA during visits (quote 1; Table 2). Assessments of the PA of people with CF were based on functional tests, medical files, initial interviews, and current and past sporting practices. Personalized PA recommendations were made according to individual objectives, preferences, and medical records. Referrals to an adapted PA professional were made for individualized care. Specific limits were set for patients with respiratory issues (quote 2; Table 2).

##### Perceptions of the CF-DB-PA Questionnaire

This category was divided into two subcategories: (1) benefits of the CF-DB-PA and (2) limitations of the CF-DB-PA. The health care professionals reported several perceived benefits of the CF-DB-PA scale. Of the 11 health care professionals, 9 (82%) provided specific information about the facilitators and barriers encountered by people with CF (quote 3; Table 2). A total of 7 (64%) health care professionals mentioned that the CF-DB-PA enabled them to evaluate the level of motivation for PA of the people with CF (quote 4; Table 2). Similarly, 7 (64%) participants mentioned that this would enable them to be aware of their patients’ level of knowledge or beliefs and help them start discussions on the subject (quote 5; Table 2). Finally, 5 (45%) health care professionals and 2 (33%) of the 6 people with CF mentioned that simply completing the CF-DB-PA would help the people with CF to think about starting or resuming PA. This is illustrated by quote 6 in Table 2.

The main perceived limitation of the scale noted by 9 (82%) health care professionals and 4 (67%) people with CF was the amount of time needed to process the results (quote 7; Table 2). A second limitation, cited by 6 (55%) health care professionals and 3 (50%) people with CF, was the “paper and pencil” version of the instrument, which does not allow responses to the questionnaire to be quickly added to the patient file and shared with the entire medical team (quote 8; Table 2).

##### Functionalities That Could Be Added to the Digital Version of the Questionnaire

This category was divided into three subcategories: (1) user-friendly interface, (2) easy data extraction, and (3) secure patient data handling. The main element that emerged while creating the digital version of the CF-DB-PA through an app was the simplicity of the interface, mentioned by 15 of the 17 participants (ie, health professionals: 10/11, 91%; people with CF: 6/6, 100%). A frequent request was to have an app that is easy to use, user-friendly, and intuitive so that the needed information can be quickly retrieved (quote 9; Table 2). The second requirement mentioned by all 11 health care professionals was easy data extraction from the digital tool for uploading to the patient file (quote 10; Table 2). The last point mentioned by almost half the participants (ie, health professionals: 7/11, 64%; people with CF: 1/6, 17%) was the importance of having a platform that guarantees the security and confidentiality of patient data (quote 11; Table 2).

##### Prospects for Using the Digital Application

This category was divided into two subcategories: (1) willingness to adopt the digital format and (2) reasons to adopt the digital format. Of the 11 health care professionals and 6 patients, only 1 (6%) patient (ie, person with CF #5) indicated that he was not inclined to use a digital app because he was not comfortable with this format (quote 12; Table 2). The other participants mentioned their intention to use the digital format for several reasons, such as to limit the use of the paper format (quote 13; Table 2). This also reflects the ubiquitous use of smartphones and other digital technologies in today’s lifestyle (quote 14; Table 2).

#### Conceptualization of the App Prototype Based on the Co-Design Meetings

##### Types of Users of the App

The initial version of the app included three user types: (1) the health care professional type, (2) the person with CF type, and (3) the researcher type. Each user type has restricted rights in the app to ensure the anonymization of the collected data.

##### Functionalities Included in the App

Drawing on the 3 co-design meetings, the specifications for the functionalities to be included in the digital app were drawn up. The functionalities were defined according to 3 levels of importance: priority, secondary, or tertiary. The priority functionalities were as follows: (1) data collection of the CF-DB-PA questionnaire, (2) intuitive visualization of the results, and (3) PA recommendations for people with CF according to the results of the questionnaire.

For the data collection, health care professionals could add new users and send the CF-DB-PA questionnaire to their associated people with CF with a request for completion. Once the questionnaires had been completed, these professionals were able to visualize the overall and subscale scores according to the nature of the barriers, in schematic and numerical forms, and extract them. The answers to each question were also available and could be extracted. A graphic also depicted the evolution of the decisional balance scores between 2 measurement times. The recommendation generated at each time was also visualized.

The people with CF were able to complete the 23-item questionnaire (Table 1) and then view and download their results. A schematic representation of their results was generated in conjunction with their PA recommendation. The evolution in their decisional balance scores between 2 measurement times could also be visualized.

Researchers could access the results of the people with CF and the visualization but without personal associated information. Researchers were not allowed to add other user accounts.

##### Development of PA Recommendations for People With CF

On the basis of the results of the CF-DB-PA questionnaire and for each of the 3 dimensions (ie, physical, psychological, and environmental), participants could have one of the three following decisional swings: (1) barrier score>facilitator score, (2) barrier score=facilitator score, and (3) barrier score<facilitator score. For each of the 9 potential situations, a specific recommendation was drawn up by the authors and is described in Table 3.

**Table 3:** Physical activity recommendations drawn by the authors for the 3 swings of the decisional balance according to the 3 dimensions of the CF-DB-PA^a^ questionnaire.

	Physical dimension	Psychological dimension	Environmental dimension
Barrier score>facilitator score	Prevalence of physical barriers. Fatigue or the symptoms of your illness are frequent obstacles to physical activity. But these barriers can be reduced by regular exercise. This is adapted so that you can exercise while taking into account the progression of your disease. You can do it, so keep on doing it!	Prevalence of psychological barriers. Lack of confidence in one’s physical abilities and apprehensions often limit the practice of physical activity. Adapted physical activity instructors propose safe situations that are accessible to everyone. This will help you to gradually regain confidence in yourself and your physical abilities. You can do it, so keep on doing it!	Prevalence of environmental barriers. Of all the physical activity options available near you, not all are suited to your needs or desires. Your local hospital can help you find the one that suits you best. You can do it, so keep on doing it!
Barrier score=facilitator score	Balance between physical barriers and facilitators. Fatigue and the symptoms of your illness are frequent obstacles to physical activity. Specialists in adapted physical activity can advise you and adapt the sessions to the progression of your illness. This will enable you to practice regularly at your own pace, and thus reduce these barriers as much as possible. You should know that the beneficial effects of physical activity are accentuated by regular exercise. You’re on the right track, so keep up the good work!	Balance between psychological barriers and facilitators. Lack of confidence in one’s physical abilities and apprehensions often limit the practice of physical activity. Adapted physical activity instructors offer safe, accessible activities for everyone. They will be able to advise you on how to exercise and adapt the sessions to your own pace. This will help you to feel more at ease in your body. You’ll find it easier to cope with the way others look at you if you’ve got someone at your side. You’re on the right track, so keep up the good work!	Balance between environmental barriers and facilitators. Of all the physical activity options available near you, not all are suited to your needs or desires. Your local hospital can help you find the one that suits you best. You’re on the right track, so keep up the good work!
Barrier score<facilitator score	Prevalence of physical facilitators. You’re aware of the benefits of physical activity for your body as a whole, and you’re managing to exercise regularly. Well done − keep up the good work!	Prevalence of psychological facilitators. You have confidence in yourself and your physical abilities. You know that physical activity is good for your health, which is why you don’t dread exercise sessions. On the contrary, they help you clear your mind and you enjoy them. Well done − keep up the good work!	Prevalence of environmental facilitators. Our local environment is conducive to physical activity. You know where to turn and where to go to exercise. Well done − keep up the good work!

^a^CF-DB-PA: Cystic Fibrosis Decisional Balance of Physical Activity.

An algorithm (ie, set of 2 rules) was created to attribute the right recommendation to the right people with CF results according to the following rules: (1) the global decisional balance score defines the swing selected, and (2) the higher score in the selected swing defines the targeted dimension.

##### Development of the First Version of the App

The technical specifications and their priorities, defined during the co-design meetings, were sent to the company chosen to develop the first version of the app. Several meetings were held between the company and the authors to ensure compliance with the technical specifications. Some test users of the recommendation selection algorithm were achieved to identify and correct any technical problems or errors.

### Acceptability Evaluation of the Mobile App

#### Characteristics of the Sample

The 103 participants involved in the acceptability evaluation phase comprised 47 (45.6%) health care professionals specialized in CF, 44 (42.7%) people with CF, and 12 (11.7%) researchers with an interest in health promotion or PA promotion research. The overall sample (ie, male: n=36, 35%; female: n=67, 65%) had a mean age of 37.92 (SD 11.07) years. The 47 health care professionals were composed of 5 (11%) physicians, 12 (26%) nurses (including coordination nurses, nursery nurses, and advanced practice nurses), 9 (13%) physical therapists, 8 (17%) adapted PA professionals, 4 (9%) dietitians, 3 (6%) psychologists, 1 (2%) pharmacist, and 5 (11%) professionals who did not specify their designation. The mean age of the health care professionals (male: n=6, 13%; female: n=41, 87%) was 38.96 (SD 9.93) years. The mean age of the people with CF (male: n=22, 50%; female: n=22, 50%) was 36.89 (SD 13.17) years. The mean age of the researchers (male: n=8, 67%; female: n=4, 33%) was 37.67 (SD 6.04) years.

Out of this sample of 103 participants, 29 (28.2%) also agreed to take part in individual semistructured interviews to further explore the acceptability of the digital app. We stopped recruiting new participants when we had no new emerging themes. No participant dropped out or refused to take part in these semistructured interviews conducted from September 2022 to January 2023 with a total duration of 1208 (mean 41.66, SD 8.03) minutes. This subsample had a mean age of 36.45 (SD 11.68) years and included 18 (62%) female participants and 11 (38%) male participants. There were 16 (67%) people with CF (female: n=8, 50%) with a mean age of 34.44 (SD 12.49) years and 13 (54%) health care professionals (female: n=10, 77%; adapted PA professionals: n=4, 31%; physical therapists: n=3, 23%; nurses: n=3, 23%; physician: n=1, 8%; dietitian: n=1, 8%; and psychologist: n=1, 8%) with a mean age of 38.92 (SD 10.55) years.

#### Quantitative Results of the Acceptability of the Mobile App

Descriptive statistics regarding the acceptability of the mobile app, the use frequency of technology-based PA interventions, and computer anxiety are provided in Table 4 for the different types of participants (ie, health professionals, people with CF, and researchers). Regression analysis showed that performance expectancy (β=.20; *P*=.03) and habit (β=.63; *P*<.001) were significant contributors to behavioral intention to use the mobile app. The other acceptability constructs were not significant (ie, for effort expectancy: β=.05, *P*=.55; for social influence: β=.04, *P*=.52; for facilitating conditions: β=.01, *P*=.90; for hedonic motivation: β=.06, *P*=.31; for price value: β=.05, *P*=.29). The acceptability constructs explained approximately 84% of the variance of behavioral intention.

**Table 4:** Acceptability of the mobile app based on the UTAUT2^a^ among people with CF^b^, health care professionals, and researchers.

			Health care professionals (n=47), mean (SD)		People with CF (n=44), mean (SD)		Researchers (n=12), mean (SD)		Total sample (N=103), mean (SD)
**UTAUT2 score^c^**									
	Overall	5.63 (0.82)		5.62 (0.82)		5.64 (0.81)		5.62 (0.81)	
	Performance expectancy	5.10 (1.45)		5.09 (1.13)		5.00 (1.65)		5.08 (1.33)	
	Effort expectancy	6.23 (0.79)		6.14 (0.94)		6.71 (0.49)		6.25 (0.84)	
	Social influence	4.84 (1.48)		5.26 (1.31)		4.50 (1.68)		4.98 (1.44)	
	Facilitating conditions	6.38 (0.61)		6.28 (0.90)		6.44 (0.87)		6.35 (0.77)	
	Hedonic motivation	5.25 (1.06)		5.21 (1.31)		5.22 (1.58)		5.23 (1.22)	
	Price value	6.74 (0.58)		6.68 (0.55)		6.78 (0.38)		6.72 (0.54)	
	Habits	4.64 (1.32)		4.49 (1.22)		4.50 (1.54)		4.56 (1.30)	
	Behavioral intention	5.15 (1.40)		4.86 (1.41)		4.83 (1.73)		4.99 (1.44)	
Use frequency of technology-based PA^d^ interventions^e^			3.40 (1.38)		2.84 (1.23)		3.27 (0.88)		3.15 (1.29)
Computer anxiety^f^			1.50 (0.83)		1.48 (0.78)		1.54 (1.05)		1.50 (0.83)

^a^UTAUT2: Unified Theory of Acceptance and Use of Technology 2.

^b^CF: cystic fibrosis.

^c^Each item of the Unified Theory of Acceptance and Use of Technology 2 (UTAUT2) scale was measured using a 7-point scale ranging from 1 (strongly disagree) to 7 (strongly agree).

^d^PA: physical activity.

^e^Use frequency of technology-based physical activity interventions was measured on a 7-point scale as follows: 1=never, 2=less than once a year, 3=once a year, 4=once a month, 5=once a week, 6=once a day, and 7=several times a day.

^f^Computer anxiety was measured on a 5-point scale ranging from 1 (not at all corresponding) to 5 (completely corresponding).

A 1-way multivariate analysis of the variance revealed no effect of the participant type on the acceptability of the mobile app (Wilks Ʌ=0.77; *F*_16,186_=1.61; *P*=.07; ηp^2^=0.12). Two 1-way univariate analyses of variance also showed no effect of the participant type on the use frequency of similar technologies (*F*_2,100_=2.27; *P*=.11; ηp^2^=0.04) or on computer anxiety (*F*_2,100_=0.02; *P*=.98; ηp^2^=0.00).p

#### Outputs From Semistructured Interviews on the Acceptability of the Mobile App

##### Overview

On the basis of the interview guide, the following three categories were studied: (1) perceptions of the app, (2) perceptions of the interface, and (3) missing functionalities. Examples of quotes representing these categories are provided in Table 5.

###### Perceptions of the App

This category covered both positive and negative perceptions of the app. Positive perceptions were cited by 27 participants (ie, 93% of the total sample) and were divided into six subcategories: (1) monitoring and individualized management of patients (11/29, 38%) such as the provision of personalized results for the patients according to their responses to the CF-DB-PA questionnaire (quote 1; Table 5); (2) awareness of facilitators and barriers (10/29, 34%), such as obstacles and levers to PA, encouraging individuals to reflect deeply on their attitudes, beliefs and experiences related to PA (quote 2; Table 5); (3) reaching a large audience (10/29, 34%), as the digital version of the CF-DB-PA can be transmitted remotely and, therefore, can be delivered to more patients simultaneously (quote 3; Table 5); (4) timesaving and user-friendliness (9/29, 31%), expressed in terms of a fluid, intuitive interface and rapid access to essential functions (quote 4; Table 5); (5) increasing motivation to exercise (9/29, 31%), noted by the “facilitators” section of the CF-DB-PA presenting a list of the benefits of PA (quote 5; Table 5); and (6) responding to a need (7/29, 24%), meaning that there is no other standardized tool for measuring the PA decisional balance in people with CF and, therefore, no other way to personalize physical exercise (quote 6; Table 5).

Negative perceptions of the app were highlighted by 11 (38%) of the 29 participants, and 60% (18/29) of the participants mentioned the absence of negative perceptions of the app (quote 7; Table 5). Negative perceptions were divided into two subcategories: (1) lack of motivation and perceived constraint (8/29, 28%), illustrated by not wanting to complete the questionnaire (quote 8; Table 5), and (2) technological constraint (5/29, 17%), such as not being comfortable using digital technologies or not wanting to use them for health care purposes (quote 9; Table 5).

###### Perceptions of the Interface

Positive and negative perceptions of the interface emerged. Positive perceptions of the interface were mentioned by 25 participants (ie, 85% of the total sample). Three subcategories were mentioned: (1) its simplicity and ease of use (23/29, 79%), illustrated by quote 10 in Table 5; (2) its design (9/29, 31%), particularly the presentation of the results in the form of a balance (quote 11; Table 5); and (3) its intuitiveness and playfulness (7/29, 24%;), as quote 12 in Table 5 shows.

Negative perceptions of the interface were cited by 5 of the 29 participants and consisted of two subcategories: (1) its inappropriate terminology (3/29, 10%), with the terms “facilitators” and “decisional balance” being put forward (quote 13; Table 5); and (2) the training required before using it (2/29, 7%), particularly to create an account, download results, or transmit a questionnaire (quote 14; Table 5).

###### Missing Functionalities

Missing functionalities of the app were identified by 13 of the 29 participants. These were classed into four categories: (1) interactions with health care professionals (7/29, 24%), such as an exchange forum with health care professionals (quote 15; Table 5); (2) interoperability with other PA apps (3/29, 10%), such as for step counting (quote 16; Table 5); (3) addition of PA content (2/29, 7%), such as videos, advice, examples of PA sessions (quote 17; Table 5); and (4) contact with sports associations and adapted PA professionals (2/29, 7%), for example, a map of adapted PA professionals (quote 18; Table 5).

##### Final Version of the App

The final version of the app was developed by adding the features discussed during the semistructured acceptability interviews. These modifications were either corrections of buggy features (eg, correcting the display of the algorithm attribution of PA recommendations for people with CF) or additions of features that did not affect the initial structure of the app and thus did not require a new evaluation of its acceptability (eg, adding the category “physical therapist” to the health care user type, adding a facility for health care professionals to search for their people with CF application members not yet associated with their account, to delete existing accounts). This final version was cleaned after user tests and is now operational. The app was named MUCO_BALAD.

**Table 5:** Representative quotes regarding the perceptions of the app, perceptions of the interface, and missing functionalities from health care professionals (n=13) and people with CF^a^ (n=16).

Themes and categories				Participants, n (%)		Quotes
**Perceptions of the app (n=29)**						
	**Positive perceptions of the app** **(n=27)**					
		Monitoring and individualized management of patients	11 (38)		Quote 1: “It can also help to identify any difficulties someone may be having, and so to create something individualized for the patient, something we hadn’t really realized we could do” (physiotherapist #3).	
		Awareness of facilitators and barriers	10 (34)		Quote 2: “I find the aspect of identifying biases for each person really interesting. It’s quite innovative in terms of everything I’ve seen, heard or read about in sports and cystic fibrosis” (person with CF #12).	
		Reach a large audience	10 (34)		Quote 3: “I think the application could enable us to offer the tool to more patients, who could do it in their room, even here in the residential unit, without necessarily needing to be with us [...] It would enable, yeah well, more patients to have access to this tool, something that is a bit more limited when we do it on an individual basis” (person with CF #19).	
		Time saving and user-friendliness	9 (31)		Quote 4: “The digital tool would make test-taking a whole lot easier and save us a lot of time, that’s for sure!” (adapted PA^b^ professional #5)	
		Increased motivation to exercise	9 (31)		Quote 5: “Some people will be able to pull themselves up by their own bootstraps, while others will use things like this to pull themselves up” (adapted PA professional #9).	
		Responding to a need	7 (24)		Quote 6: “I think it’s very good, you even wonder why it wasn’t done before” (nurse #16).	
	**Negative perceptions of the app (n=11)**					
		Absence of negative perceptions	18 (62)		Quote 7: “Not at all, no. Really, absolutely not” (person with CF #7).	
		Lack of motivation and perceived constraint	8 (28)		Quote 8: “It’s always a bit of a strain to have to answer questionnaires, even if they’re not very long” (person with CF #6).	
		Technological constraint	5 (17)		Quote 9: “From time to time we have people here, whether professionals or patients, who are not at all at ease with IT tools” (adapted PA professional #24).	
**Perceptions of the app interface (n=29)**						
	**Positive perceptions of the app interface** **(n=23, 73%)**					
		Simplicity and ease of use	23 (79)		Quote 10: “Very practical, very clear. It’s very well explained...so you don’t feel lost on the application” (adapted PA professional #8).	
		Design	9 (31)		Quote 11: “And it’s easy because we’ve got the thing with diagrams. It’s easier to visualize with graphics and everything rather than with just numbers” (person with CF #10).	
		Intuitiveness and playfulness	7 (24)		Quote 12: “It’s a lot of fun” (person with CF #26).	
	**Negative perceptions of the interface** **(n=5, 17%)**					
		Inappropriate terminology	3 (10)		Quote 13: “At first, it’s quite complicated to understand: the barriers, the figures. It’s not difficult, but at first you think: it’s not practical. But after a while you get used to it” (person with CF #20).	
		Training required	2 (7)		Quote 14: “It implies, all the same, training for each healthcare professional to say, well, that’s what you have to do if you want to add a colleague, if you want to find a patient, and so on” (physiotherapist #1).	
**Missing functionalities** **(n=13, 45%)**						
	Interaction with health care professionals		7 (24)		Quote 15: “To have more interaction, to be able to contact, to have a sort of map of nearby health professionals or associations” (person with CF #11).	
	Interoperability with other PA app		3 (10)		Quote 16: “After that, it depends on whether we can link things like watches and the like, so that we can integrate them to get the heart rate and so on” (person with CF #15).	
	Addition of PA content		2 (7)		Quote 17: “Tutorials with perhaps ‘small recovery programs.’ Because, in fact, I think that on the whole, people with CF especially need this, I think...I think for myself of small programs to get back on track” (person with CF #15).	
	Contact with sports associations and adapted physical activity professionals		2 (7)		Quote 18: “To have a sort of map of nearby health professionals or associations” (person with CF #11).	

^a^CF: cystic fibrosis.

^b^PA: physical activity.

## Discussion

### Principal Findings

The findings of this study led to the coconstruction of an app named MUCO_BALAD, designed for people with CF aged ≥18 years, health care professionals, and researchers to monitor the decisional balance for PA in people with CF. It was important to carry out a rigorous evaluation of its acceptability among these individuals to determine whether it would be an acceptable tool.

An essential quality of our study is that the app acceptability was measured using the UTAUT2 integrative model [18]. The results provided by the UTAUT2 questionnaire showed that the acceptability of the mobile app was high (ie, the scores were mainly >5) and technological anxiety was low (ie, mean scores <2). In addition, no differences were found between the user types (ie, health care professionals, patients, and researchers). These results, associated with the high explained variance in use intention, suggest that the app should be generally well accepted by different types of users.

This was also reflected in the interviews conducted with the 29 volunteer participants, who mainly expressed positive perceptions of the app that paralleled the dimensions of performance expectancy and effort expectancy of the UTAUT2 model. The categories “monitoring and individualized management of patients,” “awareness of facilitators and barriers,” and “reaching a large audience” were consistent with the “performance expectancy” category of the UTAUT2. Similarly, the identified categories “increased motivation to exercise” and “lack of motivation and perceived constraint” were congruent with the UTAUT2 model category “hedonic motivation,” and the category “responding to a need” matched with the “behavioral intention” category of the UTAUT2 model. Likewise, we observed an association of the “time saving and user-friendliness” and “simplicity and ease of use” categories with the “effort expectancy” dimension of the UTAUT2 model. Finally, “intuitiveness and playfulness” was consistent with the “habits” category from the UTAUT2, and “missing functionalities” was consistent with the UTAUT2 category “social influence.”

The use frequency of technology-based PA interventions was moderate for the 3 types of users, which can theoretically be related to the moderate score for habits in the UTAUT2. Although our results showed that habits were a major determinant of the behavioral intention to use the app, the app is not intended to be used on a daily basis but rather during quarterly check-ups; therefore, this result can be questioned. Similarly, the moderate results obtained in the “social influence,” “habits” and “behavioral intention” categories and the low results for “use frequency of technology-based PA interventions” may be explained by the observation that people with CF are oversolicited to complete questionnaires in the context of CF.

Finally, the semistructured interviews provided an overview of the perceptions of the app, perceptions of the interface, and missing functionalities for potential future users, following the categories in the interview guide. The fourth part of the guide (ie, prospects for using a digital app) asked for a general opinion of the app. Subsequently, the interview concluded with a mostly positive evaluation of the app, with only minor difficulties identified. These qualitative results are, therefore, consistent with the quantitative results.

### Limitations

This study nevertheless has a number of limitations. First, only the acceptability of the app was measured with a narrated video showing and explaining the different app functionalities. It would be interesting to assess its usability [29] in another study by actually testing the app. A high level of usability is associated with people’s commitment to taking charge of their health and, therefore, being more successful in achieving their objectives in terms of disease management and health promotion [30]. Measuring acceptability and usability are 2 distinct but related concepts in the design and evaluation of digital apps. Acceptability focuses on users’ overall perception of the app, while usability focuses on ease of use and performance. Both aspects are important for the success of a digital app, as it must be accepted by users and be easy to use to meet their needs.

Another limitation was the lack of elements that would have made the app usable on a regular basis. In practice, the app is designed for quarterly and annual visits, which leads to limited use. A suggestion put forward by several professionals and people with CF was to integrate PA content (eg, tutorials, advice, and examples of sessions), as well as ways of exchanging information with health care professionals and patient associations specializing in PA.

Participation in the semistructured acceptability interviews was voluntary. A selection bias may, therefore, have occurred, with more participants with positive perceptions participating in these interviews. In addition, the interview coding was performed by 1 researcher. Although all coding was checked by 2 other researchers, some recommendations are in favor of double coding.

### Comparison With Prior Work

This digital app for people with CF is consistent with the current development of technological support tools in the health care sector. A recent study has also sought to measure the acceptability of digital health applications for people with chronic obstructive pulmonary disease [31]. The results show positive acceptability among these 2 vulnerable populations for the use of digital health applications.

Similarly, other recent studies have examined the effectiveness of a mobile app based on the transtheoretical model to encourage people with chronic illnesses [32] to adopt healthy lifestyle behaviors (ie, PA and healthy eating). Results showed that participants using the app reported significant improvements in all areas of healthy living behaviors compared to those who received standard care. These studies suggest that digital apps based on decisional scales can be effective in promoting behavior change in a variety of contexts and for a variety of health behaviors. We can, therefore, expect that this app would be effective in promoting PA in CF, although further research is needed.

### Conclusions

This paper presents the various stages in the development of an app for PA promotion in the context of CF and reports on the measures of its acceptability to people with CF, health care professionals, and researchers specializing in CF. The qualitative and quantitative results are encouraging regarding the use of this digital tool for measuring the decisional balance for PA in people with CF in the targeted population, namely health care professionals, people with CF, and researchers.

## Abbreviations

CF: cystic fibrosis
CF-DB-PA: Cystic Fibrosis Decisional Balance of Physical Activity
CFTR: Cystic Fibrosis Transmembrane Conductance Regulator
COREQ: Consolidated Criteria for Reporting Qualitative Research
PA: physical activity
UTAUT2: Unified Theory of Acceptance and Use of Technology 2

## Data Availability

The data that support the findings of this study are available from the corresponding author upon reasonable request. They are not made publicly available because the participants were not specifically informed of the publication of the raw data.
